# Long-term trends, age-period-cohort patterns, and future projections of incident stroke with comorbid hypertension and diabetes among older adults in Shenzhen, China: a population-based surveillance study

**DOI:** 10.1186/s12877-026-08054-w

**Published:** 2026-07-28

**Authors:** Ping Xu, Zhixue Li, Ying Xu, Yunfeng Hou, Dewang Wang, Kaihao Lin, Lin Lei

**Affiliations:** 1 Department of Non-Communicable Disease Comprehensive Prevention and Control, Shenzhen Bao’an Center for Chronic Diseases Control, Shenzhen, 518000 China; 2 Shenzhen Healthcare Committee Office, Shenzhen, 518000 China; 3 Department of Cancer Prevention and Control, Shenzhen Center for Chronic Disease Control, Shenzhen, 518000 China

**Keywords:** Stroke, Hypertension, Diabetes, Multimorbidity, Age-period-cohort analysis, Bayesian age-period-cohort model, Older adults, Incidence trend

## Abstract

**Background:**

Older adults with stroke frequently have coexisting hypertension and diabetes, a high-risk cardiometabolic profile that complicates prevention, long-term management, and health service planning. However, population-based evidence on the long-term incidence trajectory of stroke occurring with both conditions remains limited, particularly in rapidly urbanizing settings undergoing population ageing. This study examined long-term trends, age-period-cohort patterns, and future projections of incident stroke with comorbid hypertension and diabetes among older adults in Shenzhen, China.

**Methods:**

This population-based retrospective surveillance study used data from the Shenzhen Cardiovascular and Cerebrovascular Disease Registry System. Incident stroke events registered between 1 January 2004 and 31 December 2023 among adults aged ≥ 60 years were identified, and 40,128 events with comorbid hypertension and diabetes were included. Crude incidence rates (CIRs) and age-standardized incidence rates (ASIRs) were calculated. Joinpoint regression, age-period-cohort analysis, and Bayesian age-period-cohort modeling were used to assess temporal trends, estimate age, period, and cohort effects, and project incidence trends from 2024 to 2033.

**Results:**

From 2004 to 2023, the number of incident stroke events with comorbid hypertension and diabetes increased from 314 to 3,336, and the ASIR rose from 141.12 per 100,000 in 2004 to 414.78 per 100,000 in 2023. Joinpoint regression showed overall increasing trends in CIR and ASIR across the study period, with average annual percent changes of 5.44% and 6.14%, respectively, while identifying a late-period decline after 2017. Incidence levels were generally higher in males than in females. Age-period-cohort analysis showed marked age effects, elevated period-related risks in recent periods, and increasing cohort effects across successive birth cohorts. Bayesian projections suggested that both CIR and ASIR may increase from 2024 to 2033, with larger projected increases in males.

**Conclusions:**

Among older adults in Shenzhen, the burden of stroke with comorbid hypertension and diabetes showed a long-term upward trajectory over the study period.

Model-based projections suggested that the burden may increase from 2024 to 2033, particularly among males and older age groups. These findings provide population-level descriptive and projection evidence that may inform risk stratification and health service planning for ageing urban populations.

**Supplementary Information:**

The online version contains supplementary material available at https://doi.org/10.1186/s12877-026-08054-w.

## Introduction

In 2021, stroke was the third leading cause of death and the fourth leading cause of disability-adjusted life-years globally [1]. Hypertension is one of the most important modifiable risk factors for stroke and remains central to primary prevention strategies [2]. Diabetes is also closely associated with stroke occurrence, recurrence, and adverse outcomes [3]. Stroke occurring in the presence of both hypertension and diabetes therefore represents a high-risk cardiometabolic condition rather than a simple aggregation of common chronic diseases. Evidence from Chinese real-world data has shown that type 2 diabetes and hypertension have both been associated with increased risks of cardiovascular and cerebrovascular events, including stroke [4]. These findings support the examination of stroke with comorbid hypertension and diabetes as a distinct cardiometabolic comorbid phenotype, rather than as part of a general comorbidity-count framework. For older adults, this phenotype is particularly important because ageing is commonly accompanied by multimorbidity, functional vulnerability, polypharmacy, and increasing demand for coordinated long-term care.

Multimorbidity has become a major challenge for ageing populations and health systems, and stroke is frequently embedded within broader cardiometabolic comorbidity patterns. In China, a recent cardiovascular health report showed that hypertension and diabetes were among the most common comorbidities among stroke inpatients, accounting for 66.5% and 27.7%, respectively [5]. A large real-world study of diabetic patients in Beijing further showed that those with stroke had a higher comorbidity burden, greater medication use, and higher related treatment costs than those without stroke [6]. Beyond clinical risk factors, international evidence shows that multimorbidity is socially patterned, with socioeconomic status, health behaviours, and contextual factors contributing to differences in disease clustering and health outcomes [7, 8]. In China, recent evidence from repeated national survey data suggests that multimorbidity among older adults has increased over time, and that its association with socioeconomic status may change alongside social and economic development [9]. These findings suggest that stroke-related cardiometabolic comorbidity should be understood not only in relation to biological ageing, but also within broader demographic, socioeconomic, and healthcare transitions.

Shenzhen provides a relevant setting for examining these patterns. As one of China’s most rapidly developed and highly urbanized cities, Shenzhen has experienced profound socioeconomic transformation over recent decades. Although the city has historically been characterized by a relatively young migrant population, recent evidence indicates that Shenzhen is undergoing accelerated and compressed population ageing with marked spatial disparities by Hukou status [10]. This demographic transition may increase demand for chronic disease prevention, primary care, and long-term health service planning. Local studies have reported a substantial burden of hypertension and type 2 diabetes among older adults in Shenzhen, with persistent gaps in treatment and control [11, 12]. Together, these demographic, socioeconomic, and cardiometabolic characteristics make Shenzhen an informative urban setting for evaluating how stroke-related cardiometabolic comorbidity evolves during rapid socioeconomic and demographic transition.

Long-term population-based evidence on incident stroke with comorbid hypertension and diabetes among older adults remains limited, particularly in rapidly urbanizing settings. Previous studies have mainly examined the overall burden of stroke [1], stroke or diabetes-related comorbidity burden [5, 6], multimorbidity and its socioeconomic patterning [7–9], or hypertension and diabetes as separate conditions among older adults [11, 12]. Few studies have characterized the long-term incidence trajectory of stroke occurring with both hypertension and diabetes. To address this gap, this study used data from the Shenzhen Cardiovascular and Cerebrovascular Disease Registry System from 2004 to 2023 to examine long-term trends, age-period-cohort patterns, and future projections of incident stroke with comorbid hypertension and diabetes among adults aged ≥ 60 years. By characterizing the temporal evolution and projected burden of this high-risk comorbid condition, this study aimed to provide evidence for risk stratification and health service planning for ageing urban populations.

## Methods

### Data source and study population

This population-based retrospective surveillance study used data from the Shenzhen Cardiovascular and Cerebrovascular Disease Registry System. Stroke events diagnosed and registered between 1 January 2004 and 31 December 2023 were identified from the registry. Case report forms included demographic characteristics, date of diagnosis, diagnostic evidence, relevant risk factors, and comorbidity information.

A total of 414,965 incident stroke events were initially identified. After excluding records with missing information on sex or age (*n* = 448; missing sex, *n* = 12; missing age, *n* = 436) and events occurring in individuals aged < 60 years (*n* = 183,699), 230,818 incident stroke events among adults aged ≥ 60 years were retained. Of these, 40,128 events with comorbid hypertension and diabetes were included in the present analysis.

Population data for permanent residents in Shenzhen were obtained from the Shenzhen Statistical Yearbook and census tabulations released by the Shenzhen Municipal Bureau of Statistics. Age- and sex-specific denominators for adults aged ≥ 60 years were available from the 2010 and 2020 national censuses. For each sex and 5-year age subgroup, annual population counts for non-census years were estimated using linear interpolation between the two census years and linear extrapolation for years outside the census interval. This approach preserved the official census counts in 2010 and 2020 and assumed constant annual change within each age-sex subgroup.

### Case definition and comorbidity ascertainment

Stroke events were classified according to the International Classification of Diseases, 10th Revision (ICD-10), including hemorrhagic stroke (I60–I62), ischemic stroke (I63), and unspecified stroke (I64) [13]. Incident stroke was defined using a 28-day episode window: recurrent events or symptom fluctuations within 28 days after stroke onset in the same patient were considered part of the same episode, whereas a new or recurrent acute event occurring more than 28 days after the previous onset was counted as a new incident event. The analysis was therefore based on incident stroke events defined by this 28-day episode window, rather than first-ever stroke events among unique individuals. Comorbid hypertension and diabetes were ascertained from information recorded in the registry case report forms at the time of stroke registration, based on documented clinical diagnoses, medical history, or information obtained during the index stroke-related clinical encounter.

Data quality was ensured through routine checks by reporting institutions, multilevel review by municipal and district chronic disease prevention and control institutions, and regular assessments of underreporting and reporting accuracy. Cross-level data exchange, including linkage with provincial surveillance systems and death registration data, was used to improve reporting completeness. The registry quality control targets required a reporting accuracy rate of at least 95% and an underreporting rate below 5%.

### Outcome measures and rate calculation

The outcomes of interest were annual event counts, crude incidence rates (CIRs), and age-standardized incidence rates (ASIRs) of incident stroke events with comorbid hypertension and diabetes among adults aged ≥ 60 years in Shenzhen. CIRs and ASIRs were calculated by calendar year, sex, and age group and expressed per 100,000 population. ASIRs were calculated by direct standardization using the WHO World Standard Population for 2000–2025 as the reference [14].

### Joinpoint regression analysis

Joinpoint regression was used to assess temporal trends in the CIRs and ASIRs over the study period. Segmented log-linear models were fitted to identify significant changes in temporal trends, with the optimal number and location of joinpoints determined using Monte Carlo permutation tests. Annual percent change (APC) and average annual percent change (AAPC) were estimated with 95% confidence intervals (CIs). Trends were considered statistically significant when the 95% CI did not include 0 [15]. Analyses were performed using the Joinpoint Regression Program, version 5.4.0.0.

### Age-period-cohort analysis

Age-period-cohort analysis was performed to examine the contributions of age, calendar period, and birth cohort effects to temporal variation in incidence. A Lexis table was constructed using 5-year age groups and 5-year calendar periods. Age was categorized as 60–64, 65–69, 70–74, 75–79, 80–84, and ≥ 85 years, and calendar period was grouped into 2004–2008, 2009–2013, 2014–2018, and 2019–2023. Birth cohorts were derived from the intersections of age group and calendar period. Given the inherent non-identifiability arising from the linear dependency among age, period, and cohort, interpretation was based on estimable functions generated by the National Cancer Institute Age-Period-Cohort Analysis Web Tool, including the longitudinal age curve, net drift, local drift, period and cohort rate ratios (RRs), and Wald test results [16].

### Bayesian age-period-cohort projection

Bayesian age-period-cohort (BAPC) models were used to project the CIRs and ASIRs from 2024 to 2033. Under a Poisson likelihood framework, age-specific event counts were modeled with the corresponding population denominators included as offsets, and Bayesian inference was conducted using integrated nested Laplace approximations (INLA) [17]. Age and birth cohort effects were modeled using second-order random walk (RW2) priors to capture smooth nonlinear patterns, whereas period effects were modeled using a first-order random walk (RW1) prior to reflect gradual temporal changes. Precision parameters were assigned weakly informative log-gamma priors with parameters 1 and 0.001. For each projection year, posterior means and 95% CIs were reported. Model fit over the observed period was assessed by comparing fitted and observed rates from 2004 to 2023 using mean absolute error (MAE), root mean square error (RMSE), mean absolute percentage error (MAPE), and 95% CI coverage. BAPC projections were performed in R version 4.2.2 using the BAPC package with INLA.

All statistical tests were two-sided, and P values < 0.05 were considered statistically significant.

## Results

### Descriptive changes in event numbers and incidence rates

A total of 40,128 incident stroke events with comorbid hypertension and diabetes among older adults were included in the analysis, including 20,989 events in males and 19,139 events in females. The mean age at stroke registration was 72.28 ± 7.95 years overall, 71.58 ± 7.86 years in males, and 73.04 ± 7.97 years in females. From 2004 to 2023, the number of incident stroke events with comorbid hypertension and diabetes among older adults in Shenzhen increased from 314 to 3,336, representing a 962.42% increase. Over the same period, both CIR and ASIR showed an overall increase. In the total population, the ASIR rose from 141.12 per 100,000 in 2004 to 414.78 per 100,000 in 2023 (Table 1).

Sex-stratified analyses showed that, overall, the number of events and incidence rates were higher in males than in females. Among males, the ASIR increased from 158.27 per 100,000 in 2004 to 474.34 per 100,000 in 2023, while among females, the corresponding ASIR increased from 125.73 per 100,000 in 2004 to 361.98 per 100,000 in 2023 (Table 1).

**Table 1:** Overall changes in incident stroke events, CIRs, and ASIRs of stroke with comorbid hypertension and diabetes among older adults in Shenzhen, China, 2004–2023

Sex	Events			CIRs per 100,000			ASIRs per 100,000		
	2004	2023	% change	2004	2023	% change	2004	2023	% change
Total	314	3336	962.42	132.81	350.04	163.57	141.12	414.78	193.93
Male	167	1818	988.62	144.98	405.81	179.91	158.27	474.34	199.71
Female	147	1518	932.65	121.24	300.56	147.90	125.73	361.98	187.91

Notes: *CIR *crude incidence rate, *ASIR *age-standardized incidence rate. % change was calculated as [(value in 2023 − value in 2004) / value in 2004] × 100

### Temporal trends based on Joinpoint regression

**Fig. 1:**
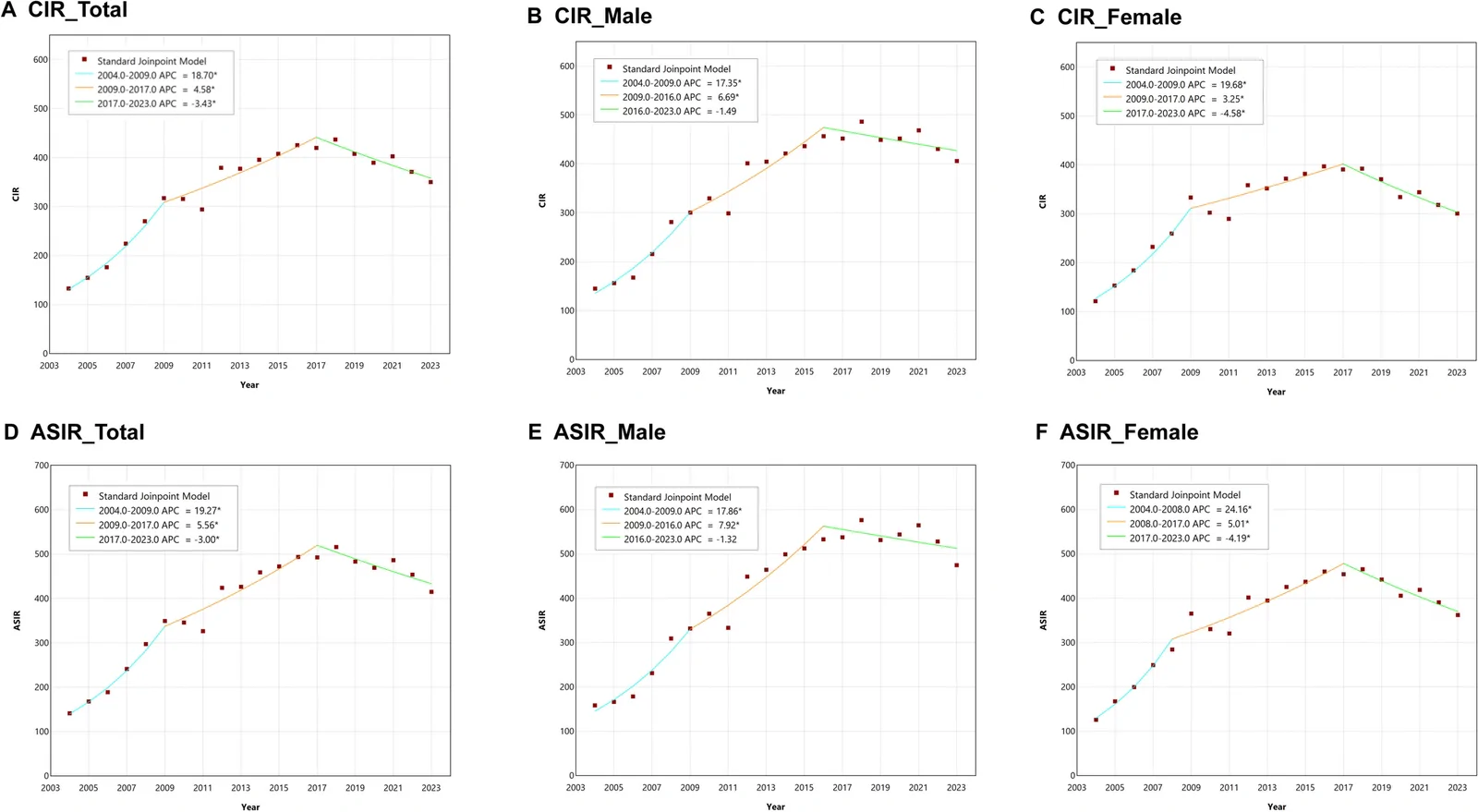
Joinpoint regression analysis of temporal trends in CIRs (**A**, Total; **B**, Male; **C**, Female) and ASIRs (**D**, Total; **E**, Male; **F**, Female) for incident stroke with comorbid hypertension and diabetes among older adults in Shenzhen, 2004–2023. Red dots represent observed rates, and colored lines represent fitted Joinpoint regression segments. An asterisk (*) indicates that APC was significantly different from zero at the 0.05 level. CIR, crude incidence rate; ASIR, age-standardized incidence rate; APC, annual percent change

Joinpoint regression showed that both CIRs and ASIRs followed a segmented temporal pattern characterized by an initial increase and a subsequent decline. In the total population, the CIR increased rapidly during 2004–2009 (APC = 18.70%, 95% CI: 14.76% to 22.78%, *P* < 0.001), continued to increase at a slower pace during 2009–2017 (APC = 4.58%, 95% CI: 2.49% to 6.71%, *P* < 0.001), and then declined significantly during 2017–2023 (APC = − 3.43%, 95% CI: −5.86% to − 0.93%, *P* = 0.012), as shown in Fig. 1A. The overall AAPC was 5.44% (95% CI: 4.05% to 6.84%, *P* < 0.001) (Table 2).

The ASIR showed a similar pattern in the total population (Fig. 1D). The APCs for 2004–2009, 2009–2017, and 2017–2023 were 19.27%, 5.56%, and − 3.00%, respectively, and the overall AAPC was 6.14% (95% CI: 4.67% to 7.62%, *P* < 0.001) (Table 2).

Sex-stratified analyses showed broadly similar temporal patterns in males and females, with a rapid increase in the early period, a slower increase in the middle period, and a decline or stabilization in the later period (Fig. 1B, C and E, F). Overall, the increase was slightly greater in males than in females. The AAPCs for CIR and ASIR were higher in males than in females: 6.23% and 6.87% in males, respectively, compared with 4.70% and 5.68% in females, respectively (all *P* < 0.001; Table 2).

**Table 2:** AAPCs for CIRs and ASIRs of stroke with comorbid hypertension and diabetes among older adults in Shenzhen, China, 2004–2023

Sex	CIRs		ASIRs	
	AAPC, % (95% CI)	*P* value	AAPC, % (95% CI)	*P* value
Total	5.44 (4.05, 6.84)	< 0.001	6.14 (4.67, 7.62)	< 0.001
Male	6.23 (4.12, 8.38)	< 0.001	6.87 (4.54, 9.24)	< 0.001
Female	4.70 (3.50, 5.92)	< 0.001	5.68 (4.07, 7.31)	< 0.001

Notes: *CIR* crude incidence rate, *ASIR *age-standardized incidence rate, *AAPC *average annual percent change, *CI *confidence interval. Estimates were obtained using Joinpoint regression models fitted separately for the total population and each sex group

### Age, period, and cohort effects on incidence

Age-period-cohort analysis showed significant age-, period-, and cohort-related patterns in the incidence of stroke with comorbid hypertension and diabetes. Wald chi-square tests showed that the main estimable functions were statistically significant in the total population and in both sexes. For cohort deviations and local drifts, the male-specific P values were 0.036 and 0.022, respectively, whereas the corresponding tests yielded *P* < 0.001 in both the total population and females (Supplementary Table S1).

**Fig. 2:**
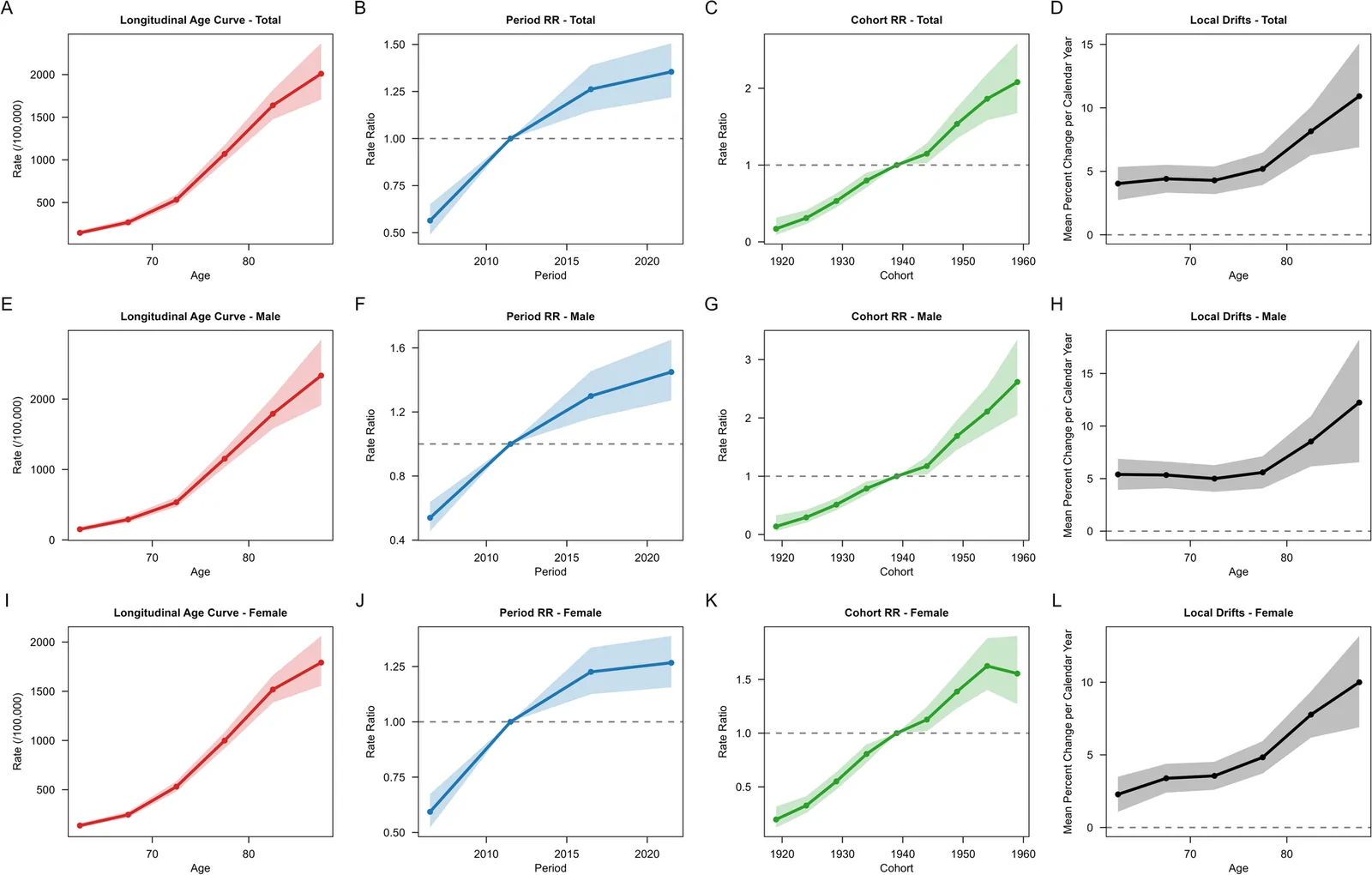
Age-period-cohort analysis of incident stroke with comorbid hypertension and diabetes among older adults in Shenzhen, China, 2004–2023. Panels **A–D** show the longitudinal age curves, period rate ratios (RRs), cohort rate ratios (RRs), and local drifts for the total population; panels **E–H** show the corresponding estimates for males; and panels **I–L** show the corresponding estimates for females. Shaded areas indicate 95% confidence intervals

Regarding age effects, the fitted incidence rate increased markedly with advancing age in the total population, rising from 105.55 per 100,000 (95% CI: 88.12 to 126.42) in the 60–64-year group to 1,415.17 per 100,000 (95% CI: 1,193.81 to 1,677.58) in the ≥ 85-year group, as shown by the longitudinal age curve in Fig. 2A. Across age groups, fitted incidence rates were generally higher in males than in females (Fig. 2E, I). Local drifts were positive across all age groups in the total population and in both sexes and generally increased with age. In the total population, local drift increased from 3.56% per year (95% CI: 2.20% to 4.94%) in the 60–64-year group to 11.28% per year (95% CI: 6.96% to 15.77%) in the ≥ 85-year group. The corresponding estimates increased from 5.08% per year (95% CI: 3.42% to 6.76%) to 12.66% per year (95% CI: 6.17% to 19.53%) in males and from 1.65% per year (95% CI: 0.35% to 2.97%) to 10.30% per year (95% CI: 6.83% to 13.88%) in females (Fig. 2D, H, and L; Supplementary Table S2).

Using 2009–2013 as the reference period (RR = 1.00), the period RR in the total population was lower in 2004–2008 (RR = 0.58, 95% CI: 0.50 to 0.67), increased to 1.31 (95% CI: 1.18 to 1.45) in 2014–2018 and remained elevated at 1.33 (95% CI: 1.19 to 1.49) in 2019–2023, as shown in Fig. 2B. In males, the period RR increased from 1.37 (95% CI: 1.21 to 1.56) in 2014–2018 to 1.46 (95% CI: 1.26 to 1.70) in 2019–2023. In females, the corresponding RR increased to 1.25 (95% CI: 1.14 to 1.37) in 2014–2018 and then declined slightly to 1.21 (95% CI: 1.10 to 1.34) in 2019–2023 (Fig. 2F, J; Supplementary Table S2).

Using the 1935–1939 birth cohort as the reference (RR = 1.00), cohort RRs generally increased across successive birth cohorts. In the total population, the RR increased from 0.17 (95% CI: 0.09 to 0.32) in the 1915–1919 cohort to 1.92 (95% CI: 1.53 to 2.41) in the 1955–1959 cohort (Fig. 2C). Sex-stratified analyses showed broadly similar upward patterns. In males, the RR increased from 0.13 (95% CI: 0.05 to 0.36) in the 1915–1919 cohort to 2.48 (95% CI: 1.88 to 3.27) in the 1955–1959 cohort. In females, the RR increased from 0.19 (95% CI: 0.11 to 0.33) in the 1915–1919 cohort to 1.51 (95% CI: 1.29 to 1.77) in the 1950–1954 cohort and then declined slightly to 1.39 (95% CI: 1.12 to 1.73) in the 1955–1959 cohort (Fig. 2G, K). Detailed age-period-cohort estimates are provided in Supplementary Table S2.

### Projected incidence trends from BAPC analysis

**Fig. 3:**
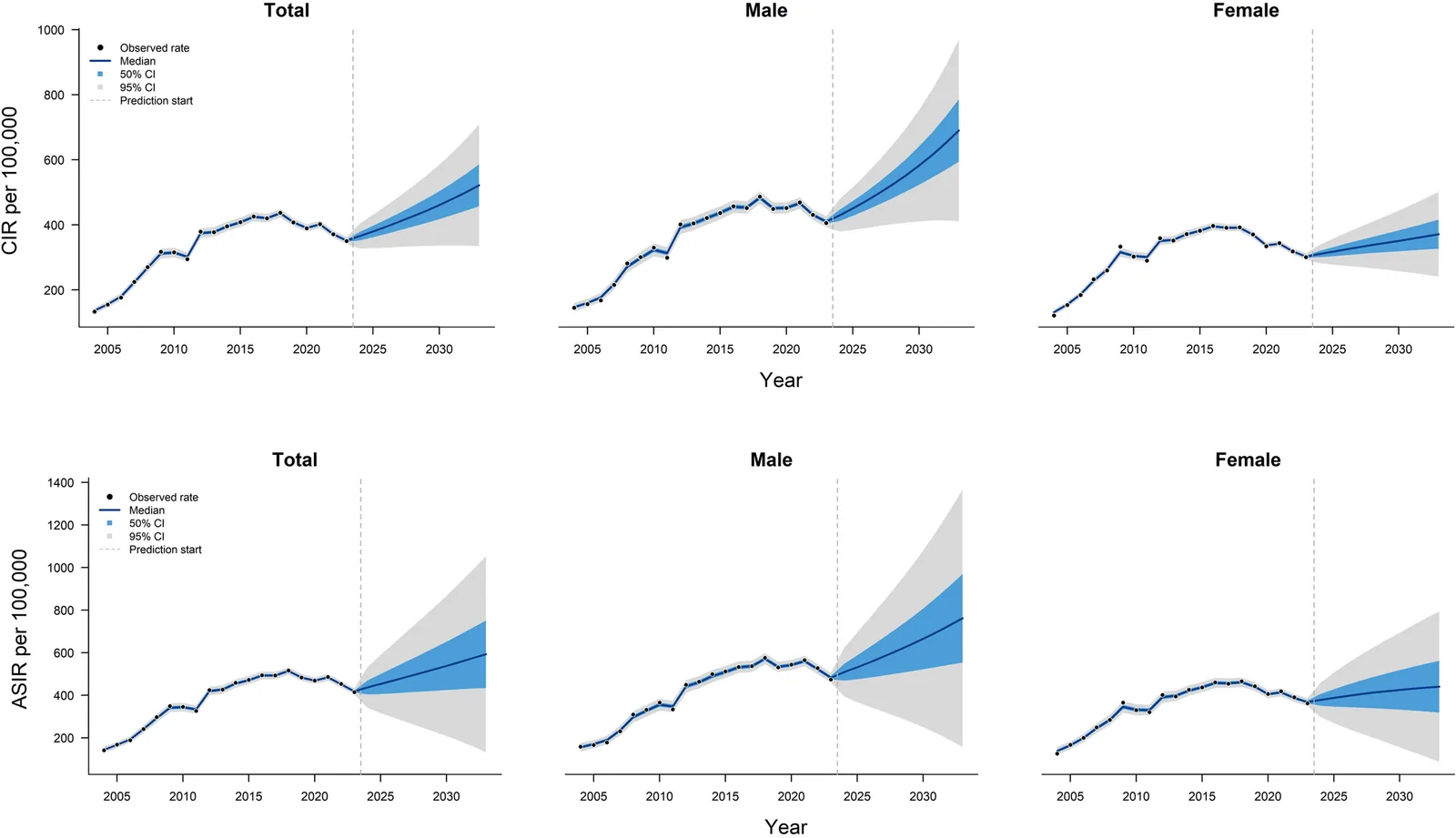
Bayesian age-period-cohort projections of CIRs (**A**, Total; **B**, Male; **C**, Female) and ASIRs (**D**, Total; **E**, Male; **F**, Female) for incident stroke with comorbid hypertension and diabetes among older adults in Shenzhen, China, 2004–2033. Solid lines indicate median projected rates, shaded areas indicate 50% and 95% confidence intervals, and the vertical dashed line marks the start of the projection period. CIR, crude incidence rate; ASIR, age-standardized incidence rate

The BAPC model projected continued increases in both CIR and ASIR for incident stroke with comorbid hypertension and diabetes among older adults in Shenzhen from 2024 to 2033. In the total population, the projected CIR increased from 365.57 (95% CI: 327.39 to 403.74) per 100,000 in 2024 to 521.80 (95% CI: 334.55 to 709.05) per 100,000 in 2033 (Fig. 3A), while the projected ASIR increased from 436.86 (95% CI: 342.43 to 531.28) per 100,000 to 593.22 (95% CI: 132.51 to 1053.93) per 100,000 over the same period (Fig. 3D).

Sex-stratified projections showed that both CIR and ASIR remained higher in males than in females, with larger projected increases in males. In males, the projected CIR increased from 429.12 (95% CI: 379.76 to 478.48) per 100,000 in 2024 to 690.23 (95% CI: 410.98 to 969.48) per 100,000 in 2033 (Fig. 3B), and the projected ASIR increased from 508.13 (95% CI: 394.54 to 621.73) per 100,000 to 761.79 (95% CI: 156.85 to 1366.72) per 100,000 (Fig. 3E). In females, the projected CIR increased from 310.14 (95% CI: 282.46 to 337.82) per 100,000 in 2024 to 371.27 (95% CI: 241.16 to 501.38) per 100,000 in 2033 (Fig. 3C), and the projected ASIR increased from 378.23 (95% CI: 296.82 to 459.63) per 100,000 to 440.35 (95% CI: 87.32 to 793.37) per 100,000 (Fig. 3F).

## Discussion

Using population-based surveillance data from Shenzhen during 2004–2023, this study showed that the burden of stroke with comorbid hypertension and diabetes among older adults increased substantially over the long term, as reflected by rising event counts, CIRs, and ASIRs, despite a decline in both incidence measures during the final Joinpoint segment after 2017. Consistent with the historical age-period-cohort structure, BAPC projections suggested that this burden may continue to increase over the next decade. These findings characterize the changing population-level burden of a high-risk comorbid stroke phenotype in a rapidly ageing urban population.

The long-term increase in incident stroke with comorbid hypertension and diabetes among older adults in Shenzhen likely reflects the combined influence of accumulated cardiometabolic risk and incomplete risk-factor control over time. Although population ageing contributed to the rising number of events, the sustained increase in ASIR suggests that changes in age structure alone cannot fully explain the observed trend. Hypertension remains one of the most important modifiable risk factors for stroke, while diabetes is closely linked to cardiovascular and cerebrovascular risk; however, gaps in awareness, treatment, and control persist in China [2, 18, 19]. The coexistence of hypertension and dysglycemia may further amplify cardiovascular and cerebrovascular risk [4, 20]. Local evidence from Shenzhen also indicates suboptimal hypertension treatment and control among older adults, as well as a high prevalence and inadequate control of type 2 diabetes in the older population [11, 12, 21]. Taken together, this evidence provides a plausible background for the long-term increase in this high-risk comorbid stroke phenotype among older adults.

The age-period-cohort findings further support this interpretation. Incidence increased markedly with advancing age, consistent with an important contribution of population ageing to the increasing burden of stroke with comorbid hypertension and diabetes [22]. This pattern may be related to age-associated vascular ageing, endothelial dysfunction, and worsening insulin resistance [23, 24], which may in turn impair vascular and metabolic regulation and increase susceptibility to stroke in the presence of cardiometabolic comorbidity.

Within the APC framework, the period RRs further suggest that shared population-level influences continued to shape the incidence of stroke with comorbid hypertension and diabetes during the middle and later phases of the study period. The elevated period RRs may reflect both the accumulation of cardiometabolic risk factors and improvements in detection, diagnosis, and registry reporting over time [18, 25–27]. Notably, the male period RR increased from 1.37 in 2014–2018 to 1.46 in 2019–2023, whereas Joinpoint regression identified a decline in annual male incidence rates after 2017. These findings are not directly contradictory: period RRs compare relative incidence levels across broad 5-year periods with the 2009–2013 reference period, whereas Joinpoint regression estimates annual changes in the observed rate trajectory. Thus, the period RR for 2019–2023 could remain elevated while annual incidence rates declined during the later Joinpoint segment.

The upward cohort pattern across successive birth cohorts suggests that later-born cohorts may have experienced longer cumulative exposure to urbanization-related lifestyle and metabolic risks, including sedentary lifestyles, dietary change, overweight and obesity, and a higher risk of metabolic syndrome [26, 28]. Such cumulative exposures may have contributed to a higher incidence of stroke with coexisting hypertension and diabetes in later life.

Although the overall burden increased substantially over the two-decade study period, Joinpoint analysis identified a decline in both CIR and ASIR after 2017. This apparent inflection occurred during a period in which Shenzhen was strengthening chronic disease prevention, integrated primary care, and stroke care systems, including citywide coverage of the National Demonstration Areas for Integrated Chronic and Non-Communicable Disease Prevention and Control by 2017, expanded community-based management and family physician services, and development of stroke emergency care systems [27, 29–32]. These system-level developments provide relevant contextual information for interpreting the temporal pattern, but the ecological design of this study precludes direct attribution of the post-2017 decline to these changes. In addition, the COVID-19 pandemic may have affected care-seeking behaviour, healthcare access, and case ascertainment during 2020–2022, particularly for transient ischemic attack and mild stroke [33, 34]. Therefore, the post-2017 decline should be interpreted cautiously as a late-period fluctuation within an overall increasing long-term burden, rather than as evidence of a sustained reversal of the underlying trend.

The projected increase over the next decade suggests that the late-period decline should be interpreted within the broader context of the long-term upward trajectory. Because BAPC projections are informed by the historical age-period-cohort structure, the marked age gradient, broader upward cohort pattern, and continued population ageing may collectively contribute to a higher projected burden despite the decline observed during the final study segment. Persistent cardiometabolic risk accumulation may further sustain this burden in the coming years [26, 35].

At the planning level, the observed trajectory and projected burden support population-level risk stratification and health service planning for ageing urban populations. In particular, the results support consideration of integrated screening for hypertension and diabetes, long-term follow-up of older adults with both conditions, and continuous community-based monitoring of high-risk older adults [36, 37]. Strengthening coordination among family physician services, integrated primary care, and stroke emergency care networks may also support continuity of care for older adults with cardiometabolic multimorbidity [27, 32, 38]. Given the aggregated design, these implications should be interpreted as planning considerations rather than as evidence of intervention effectiveness.

Sex-stratified analyses showed that males generally had higher incidence levels and steeper upward trends in stroke with comorbid hypertension and diabetes than females, and the projected sex gap may widen further over time. This finding is broadly consistent with the sex-specific distribution of stroke burden reported in China [39]. Behavioural risk exposure may be an important contributor, as smoking and alcohol use are substantially more common among Chinese males than females [40, 41]. Unhealthy lifestyle factors may also interact with elevated blood pressure to increase stroke risk, with smoking potentially further amplifying this effect [42]. In addition, blood pressure levels and hypertension burden are generally higher in Chinese males than females [43], whereas females tend to have higher levels of hypertension awareness, treatment, and control, and diabetes awareness has also been reported to be higher among females in some Chinese populations [44, 45]. These behavioural, cardiometabolic, and management-related differences may jointly contribute to the higher burden and faster increase observed in males. This sex-specific pattern supports consideration of male-focused behavioural risk reduction and cardiometabolic risk monitoring in future prevention planning, although individual-level intervention effects were not evaluated in this study.

This study has several strengths. First, the long study period allowed a comprehensive characterization of the long-term trajectory of incident stroke with comorbid hypertension and diabetes among older adults in Shenzhen. Second, this study was based on a population-based surveillance system, enabling the assessment of temporal patterns in a large number of incident stroke events over two decades. Third, by integrating event counts, CIRs, and ASIRs and combining Joinpoint regression, age-period-cohort analysis, and BAPC modeling, this study provided a systematic assessment spanning trend identification, age-period-cohort pattern characterization, and future projection. This analytical framework helped characterize both the historical evolution and projected burden of this high-risk comorbid stroke phenotype.

Several limitations should be acknowledged. First, the surveillance data relied on routine clinical diagnosis and reporting, and underreporting, particularly of nonfatal and mild stroke, may have occurred. Ascertainment of hypertension, diabetes, and comorbid status may also have been influenced by healthcare access, diagnostic practices, and medical-record completeness. Improvements in diagnosis, documentation, and reporting over time may therefore have increased comorbidity detection and led to some overestimation of the observed long-term increase. Second, stroke subtypes were not analysed separately, although ischemic, hemorrhagic, and unspecified stroke may differ in etiological pathways and temporal trends. Third, the aggregated data did not include individual-level behavioural, metabolic, socioeconomic, treatment-related, or healthcare utilization factors, limiting causal inference regarding underlying mechanisms. Finally, APC analyses are constrained by the inherent non-identifiability of the independent linear effects of age, period, and cohort. The use of four calendar periods and six age groups may also have limited period–cohort separation and increased uncertainty in edge-cohort estimates, particularly among males. BAPC projections assume continuity of historical patterns, and the population denominator estimates were also subject to uncertainty.

## Conclusion

In this population-based surveillance study, the burden of stroke with comorbid hypertension and diabetes among older adults in Shenzhen showed a substantial long-term increase from 2004 to 2023, despite a decline during the final Joinpoint segment after 2017. Model-based projections suggested that this burden may continue to increase through 2033, particularly among males and older age groups. These population-level descriptive and projection findings may inform risk stratification, integrated hypertension-diabetes screening and follow-up, male-focused behavioural risk reduction, and health service planning for rapidly ageing urban populations.

## Supplementary Information

Supplementary Material 1: Supplementary Table S1. Wald Chi-square tests for estimable parameters in the age-period-cohort model.

Supplementary Material 2: Supplementary Table S2. Age-period-cohort estimates for incident stroke with comorbid hypertension and diabetes among older adults in Shenzhen, China, 2004–2023.

## Data Availability

The data that support the findings of this study are derived from the Shenzhen Cardiovascular and Cerebrovascular Disease Registration and Reporting System. Owing to data protection regulations and restrictions imposed by the data provider, the datasets are not publicly available. Access to the data may be granted upon reasonable request and subject to approval by the relevant authorities.

## Abbreviations

CIR: Crude incidence rate
ASIR: Age-standardized incidence rate
BAPC: Bayesian age–period–cohort
CI: Confidence interval
APC: Annual percent change
AAPC: Average annual percent change
RR: Rate ratio
INLA: Integrated nested Laplace approximations
RW2: Second-order random walk
RW1: First-order random walk
MAE: Mean absolute error
RMSE: Root mean square error
MAPE: Mean absolute percentage error
