# Supplementary material for: Long-term trends, age-period-cohort patterns, and future projections of incident stroke with comorbid hypertension and diabetes among older adults in Shenzhen, China: a population-based surveillance study
Source: BMC Geriatr. 2026 Jul 28;26:1343. doi: 10.1186/s12877-026-08054-w (PMC13700160; doi:10.1186/s12877-026-08054-w)
Supplement: Supplementary file 1 [file 12877_2026_8054_MOESM1_ESM.docx]

**Supplementary Table S1.** Wald Chi-square tests for estimable parameters in the age-period-cohort model

| Waldtests | Total | |  | Male | |  | Female | |
| --- | --- | --- | --- | --- | --- | --- | --- | --- |
|  | χ² | P-Value |  | χ² | P-Value |  | χ² | P-Value |
| NetDrift = 0 | 136.24 | <0.001 |  | 99.96 | <0.001 |  | 124.90 | <0.001 |
| All Age Deviations = 0 | 55.24 | <0.001 |  | 29.01 | <0.001 |  | 84.56 | <0.001 |
| All Period Deviations = 0 | 43.77 | <0.001 |  | 27.11 | <0.001 |  | 52.52 | <0.001 |
| All Cohort Deviations = 0 | 27.95 | 0.001 |  | 15.04 | 0.036 |  | 46.99 | <0.001 |
| All Period RR = 1 | 146.00 | <0.001 |  | 106.16 | <0.001 |  | 138.28 | <0.001 |
| All Cohort RR = 1 | 153.05 | <0.001 |  | 123.01 | <0.001 |  | 148.68 | <0.001 |
| All Local Drifts = Net Drift | 27.16 | <0.001 |  | 14.78 | 0.022 |  | 45.44 | <0.001 |
