# Supplementary material for: Long-term trends, age-period-cohort patterns, and future projections of incident stroke with comorbid hypertension and diabetes among older adults in Shenzhen, China: a population-based surveillance study
Source: BMC Geriatr. 2026 Jul 28;26:1343. doi: 10.1186/s12877-026-08054-w (PMC13700160; doi:10.1186/s12877-026-08054-w)
Supplement: Supplementary file 2 [file 12877_2026_8054_MOESM2_ESM.docx]

**Supplementary Table S2.** Age-period-cohort estimates for incident stroke with comorbid hypertension and diabetes among older adults in Shenzhen, China, 2004–2023

| Sex | Longitudinal age curves | | Period RRs | | Cohort RRs | | Local Drifts | |  |
| --- | --- | --- | --- | --- | --- | --- | --- | --- | --- |
|  | Age group,  years | Rate per 100,000  (95% CI) | Period | RR (95% CI) | Birth cohort | RR (95% CI) | Age group,  years | Local drift, % per year (95% CI) |  |
| Total | 60-64 | 105.55 (88.12, 126.42) | 2004-2008 | 0.58 (0.50, 0.67) | 1915-1919 | 0.17 (0.09, 0.32) | 60-64 | 3.56 (2.20, 4.94) |  |
|  | 65-69 | 198.99 (172.89, 229.04) | 2009-2013 | 1.00 (reference) | 1920-1924 | 0.31 (0.23, 0.42) | 65-69 | 4.06 (2.93, 5.21) |  |
|  | 70-74 | 392.79 (350.71, 439.92) | 2014-2018 | 1.31 (1.18, 1.45) | 1925-1929 | 0.55 (0.46, 0.65) | 70-74 | 3.97 (2.86, 5.09) |  |
|  | 75-79 | 789.79 (717.17, 869.77) | 2019-2023 | 1.33 (1.19, 1.49) | 1930-1934 | 0.81 (0.72, 0.92) | 75-79 | 4.90 (3.59, 6.23) |  |
|  | 80-84 | 1199.89 (1075.08, 1339.18) |  |  | 1935-1939 | 1.00 (reference) | 80-84 | 8.16 (6.17, 10.18) |  |
|  | ≥85 | 1415.17 (1193.81, 1677.58) |  |  | 1940-1944 | 1.13 (1.01, 1.28) | ≥85 | 11.28 (6.96, 15.77) |  |
|  |  |  |  |  | 1945-1949 | 1.49 (1.30, 1.71) |  | |  |
|  |  |  |  |  | 1950-1954 | 1.77 (1.50, 2.10) |  |  |  |
|  |  |  |  |  | 1955-1959 | 1.92 (1.53, 2.41) |  |  |  |
| Male | 60-64 | 106.81 (85.54, 133.36) | 2004-2008 | 0.56 (0.46, 0.68 ) | 1915-1919 | 0.13 (0.05, 0.36 ) | 60-64 | 5.08 (3.42, 6.76) |  |
|  | 65-69 | 209.83 (176.28, 249.76) | 2009-2013 | 1.00 (reference) | 1920-1924 | 0.29 (0.19, 0.43 ) | 65-69 | 5.19 (3.78, 6.62) |  |
|  | 70-74 | 379.77 (329.36, 437.90) | 2014-2018 | 1.37 (1.21, 1.56) | 1925-1929 | 0.52 (0.42, 0.66) | 70-74 | 4.84 (3.45, 6.25) |  |
|  | 75-79 | 842.71 (746.52, 951.29) | 2019-2023 | 1.46 (1.26, 1.70) | 1930-1934 | 0.80 (0.68, 0.94 ) | 75-79 | 5.39 (3.72, 7.08) |  |
|  | 80-84 | 1296.61 (1126.45, 1492.47) |  |  | 1935-1939 | 1.00 (reference) | 80-84 | 8.73 (6.03, 11.50) |  |
|  | ≥85 | 1641.24 (1316.68, 2045.81) |  |  | 1940-1944 | 1.17 (1.01, 1.35) | ≥85 | 12.66 (6.17, 19.53) |  |
|  |  |  |  |  | 1945-1949 | 1.67 (1.41, 1.98 ) |  |  |  |
|  |  |  |  |  | 1950-1954 | 2.06 (1.67, 2.53) |  |  |  |
|  |  |  |  |  | 1955-1959 | 2.48 (1.88, 3.27) |  |  |  |
| Female | 60-64 | 103.67 (87.54, 122.79) | 2004-2008 | 0.60 (0.53, 0.69) | 1915-1919 | 0.19 (0.11, 0.33 ) | 60-64 | 1.65 (0.35, 2.97) |  |
|  | 65-69 | 187.95 (164.92, 214.18) | 2009-2013 | 1.00 (reference) | 1920-1924 | 0.33 (0.26, 0.43 ) | 65-69 | 2.85 (1.79, 3.91) |  |
|  | 70-74 | 406.43 (366.41, 450.82) | 2014-2018 | 1.25 (1.14 ,1.37 ) | 1925-1929 | 0.57 (0.49, 0.67 ) | 70-74 | 3.08 (2.06, 4.11) |  |
|  | 75-79 | 744.50 (681.62, 813.19) | 2019-2023 | 1.21 (1.10, 1.34） | 1930-1934 | 0.82 (0.73, 0.92 ) | 75-79 | 4.46 (3.28, 5.65) |  |
|  | 80-84 | 1122.73 (1017.22, 1239.18) |  |  | 1935-1939 | 1.00 (reference) | 80-84 | 7.61 (5.91, 9.34) |  |
|  | ≥85 | 1260.30 (1083.12, 1466.45) |  |  | 1940-1944 | 1.11 (0.99, 1.23 ) | ≥85 | 10.30 (6.83, 13.88) |  |
|  |  |  |  |  | 1945-1949 | 1.32 (1.16,1.50) |  |  |  |
|  |  |  |  |  | 1950-1954 | 1.51 (1.29, 1.77) |  |  |  |
|  |  |  |  |  | 1955-1959 | 1.39 (1.12, 1.73) |  |  |  |

Notes: Longitudinal age curves are expressed as incidence rates per 100,000 population. Period and cohort effects are presented as rate ratios (RRs) with 95% confidence intervals (CIs). Local drifts are presented as annual percent changes with 95% CIs. The reference period was 2009–2013, and the reference birth cohort was 1935–1939.
